# Fracture Detectives: A Fracture Review Match Game

**DOI:** 10.21980/J8F06W

**Published:** 2020-01-15

**Authors:** Gabriel Sudario, Gina Hana

**Affiliations:** * University of California, Irvine, Department of Emergency Medicine, Orange, CA

## Abstract

**Audience:**

The target audience for this small group session is emergency medicine residents, primarily for use in didactic conference. This session can also be utilized with medical students or faculty looking to review various orthopedic injuries.

**Introduction:**

The Model of Clinical Practice of Emergency Medicine specifies content for American Board of Emergency Medicine certification and requires proficiency in a wide breadth of medical topics, including upper extremity and lower extremity orthopedic injuries.^1^ Traditional teaching sessions regarding orthopedic injuries usually rely on standard didactic presentations of injury description followed by review of imaging interpretations and management pearls. We present a novel use of gamification to tap into collective group knowledge to identify common orthopedic injuries. Our session then relies on the flipped classroom model, where learners teach relevant material to the rest of the cohort.

**Educational Objectives:**

At the end of this session, learners will be able to: recognize and identify various orthopedic injuries on plain film images, describe the mechanism of injury of the various orthopedic injuries, describe the physical examination findings seen in various orthopedic injuries, recall associated injuries and at-risk anatomic structures associated with various orthopedic injuries, and describe the emergency department management of various orthopedic injuries.

**Educational Methods:**

This session is grounded in two educational methods, gamification and the flipped classroom model. Gamification is implemented by being modeled after the popular group game, “Who Am I?” Learners are randomly given a paper card that has printed either the name of a common orthopedic injury or X-ray image of that injury. These cards are taped to the learner’s back, without learners being aware of the diagnosis they are in possession of. By asking yes or no questions to others in the room, learners attempt to identify their specific diagnosis and find the pair that he or she matches with in the room. The educational strategy of flipped-classroom comes into play after all pairs are identified. Learners work in these paired groups to prepare one digital slide teaching the salient points related to their diagnosis. Learners all work on a shared Google Drive Slides document and present the material to the entire group at the end of the session.

**Research Methods:**

Educational content and satisfaction were obtained from learners through in-person interviews at the end of the session. Learners were asked questions regarding relevance, satisfaction with structure of the session, and overall value of the session related to their clinical practice.

**Results:**

Overall, residents had high levels of satisfaction after the session, many commenting on how gamification made the session more interactive and interesting. Learners did give feedback regarding needing more time to complete the flipped classroom component of the session, and overall felt like their parts of the presentation were rushed.

**Discussion:**

Gamification and flipped classroom learning strategies were effective in teaching the identification and management of common orthopedic injuries. Gamification increased engagement. Flipping the classroom allowed learners to obtain deeper knowledge in one specific diagnosis while learning collectively from the knowledge of an entire cohort.

**Topics:**

Extremity bony trauma, dislocations/subluxations, tendon injuries, ligamentous injuries.

## USER GUIDE

[Table t1-jetem-5-1-sg36]

**Table t1-jetem-5-1-sg36:** 

List of Resources:	
Abstract	36
User Guide	38
Instructor Materials	42

**Learner Audience:**

Medical Students, Junior Residents, Senior Residents

**Time Required for Implementation:**

We recommend 60–90 minutes for implementation of this game, with 15 minutes of preparation prior to the session.

**Recommended Number of Learners per Instructor**: One instructor can facilitate four groups with up to fifteen participants in each group.

**Topics:**

Extremity bony trauma, dislocations/subluxations, tendon injuries, ligamentous injuries.

**Objectives:**

By the end of this session, learners will be able to:

1. Recognize and identify the diagnoses listed below on plain film imaging.
2. Describe the mechanism of injury of the diagnoses listed below.
3. Describe the physical examination findings seen in listed diagnoses below.
4. Recall associated injuries and at-risk anatomic structures associated with the diagnoses listed below.
5. Describe the emergency department management of the diagnoses listed below.

**Objective Diagnoses** ***:***

Posterior shoulder dislocation, clavicle fracture, Bennett fracture, lunate dislocation, scapholunate dissociation, perilunate dislocation, Monteggia fracture, Galeazzi fracture, tibial plateau fracture, intertrochanteric fracture, knee dislocation, Maisonneuve fracture, Jones fracture, and Lisfranc injury.

### Linked objectives and methods

Our goals and objectives are linked to two educational strategies, gamification and the flipped-classroom model. Both of these educational strategies are grounded in constructivist learning theory, which states that “knowledge is constructed by learners as they attempt to make sense of experiences” and focuses on the importance of complex situations and social negotiation where “collaboration allows insights and solutions to arise synergistically.” 3 Our use of gamification relies on group knowledge, collaboration, social negotiation, and creating a learning environment that encourages curiosity. The game sparks engagement and curiosity and requires learners to interact with other learners and facilitators. After students have found their match, flipped classroom is implemented by encouraging learners to use open resources to become knowledgeable in their specific diagnosis, learning by discovery. Learners then are expected to model and explain their approach to a specific injury to the entirety of the class. Overall, these methods provide learning strategies that align well with our objectives and the constructivist model of learning.

In terms of session content, the EM Model of Clinical Practice was reviewed and cross-referenced with the Orthopedic Teaching Database from Northwestern University to select various orthopedic injuries commonly encountered in the emergency department setting or of high morbidity if missed on evaluation.^1^,^2^ It was essential that the injury be easy to visually identify when printed on a half-page, which limited some potential injuries to review. Additional diagnoses could be easily added to this session depending on audience size.

### Recommended pre-reading for facilitator

- Facilitators should read the case descriptions corresponding with the diagnoses listed below from the Northwestern Emergency Medicine Orthopedic Teaching website.^2^ This is an open source, well-referenced, case-based orthopedic injury learning tool that provides a broad overview of each diagnosis. We also recommend facilitators read our answer key in full to become familiar with each diagnosis.

### Results and tips for successful implementation

This session was tested on 22 resident and medical student learners during a weekly emergency medicine didactic conference. Due to falling behind in conference schedule, we were only given 30 minutes for the entire session. Post-session evaluations were emailed to participants before and after the session, but no participating learners completed this survey. We did ask for verbal feedback from learners at the end of the session. Overall, learners felt that the session was entertaining, engaging, and useful in reviewing common, high morbidity orthopedic injuries. They felt that the use of a shared Google Drive slide deck was innovative. Residents did provide formative feedback regarding the session. They felt that creation of their slide felt very rushed and they did not have enough time to find high quality sources and format a slide in an ideal manner within 10 minutes. They also felt that their time to present was rushed. An unanticipated consequence of the collective work on a slide presentation was that many learners stated that when they were presenting, others were distracted by attempting to finish their slides in time for their presentation. We suggest the following best practices for improved implementation of this session:

- **Preparation:** This session is best implemented with timely preparation of game materials and digital shared slides. The name and image cards must be cut and ideally pre-loaded with a piece of adhesive tape prior to the session. Instructors must be aware of the exact number of learners participating in the exercise and provide the correct number of matched cards for learners to use during the session.
  Learners must be informed prior to the session that they should bring a laptop or tablet to the session. The shared digital slide should be pre-formatted to be editable by any person who has the link. There should be an easy mechanism to distribute the link to the slides; we used a custom link that was shortened, then placed the link in our slide deck and also wrote it on our white board.
- **Timing:** We recommend five minutes for game explanation and 15 minutes for game play. For smaller groups of learners, we recommend breaking game play into multiple rounds and repeating until all injuries are reviewed. After learners have found their correct pair, we recommend asking learners to sit down or move away from the playing field. For slide creation, we recommend 20 minutes for learners to complete their slide. Most importantly, learners should all stop working on their slides after these 20 minutes, with devices closed, so that all learners can be mindfully present during peer slide presentations.
- **Shared Slides:** It is important that the slide deck be refreshed after all learners have completed their additions to the presentation. Otherwise, the slides will still remain empty while presenting to the entire room of learners.

### Pearls

Learning summary will be available in the shared Google Slides, to which all learners will have access. At the end of the session, the instructor should read through, edit, and standardize formatting of the slides so that learners can reference them in the future.

## INSTRUCTOR MATERIALS

### Fracture Detectives: A Fracture Review Match Game

#### Small Group Application Exercise (sGAE) Instructions

##### Game Preparation (10 minutes)

Instructors will need to cut image/diagnoses cards prior to the session. Instructors should count the number of participants for the session. Instructors will then ensure there are a correct corresponding number of playing cards available for the game. It is vital that all cards have a matching corresponding diagnosis/image card in play. Lay all images/diagnoses face down on a table, ideally with a small piece of adhesive attached to each card. Image cards are available as a supplemental PDF entitled sGAE. All images are from Radiopedia, which falls under Creative Commons use (specifically, CC BY-NC-SA 3.0). All references are printed at the bottom of every image.

##### Game Explanation (5 minutes)

Explain to learners the game play. We recommend saying: “Lying on the table in front of you are X pairs of playing cards. They contain either a plain film image of an orthopedic injury or a printed name of an orthopedic injury. When I say so, please grab one card from the table and tape it on the back of **someone else** playing in the room. It is vital that this person **does not** know the image or word printed on his/her back. Once everyone has a card taped to their back, we will start game play. The goal of the game is to find the partner who matches either the diagnosis or image taped to your back. To do this, begin to ask others playing yes or no questions regarding your diagnosis. Example questions include, “Am I an injury of the upper extremity?” or “Do I require reduction?” You can only ask a participant one question and must move on to another participant to have another question answered. When you think you have found your partner, both participants will approach me and ask if they are matched correctly. If you are a correct match, sit down with your partner. If not, continue game play. It is OK to pull up resources on your phone/computer if you are asked a question that you don’t know the answer to.”

##### Game Play (15 minutes)

Allow learners to begin game play. Monitor the room and remind learners to use yes/no questions only and remind them to ask questions of multiple participants in the room. Once a player pair feels that they have found their match, confirm if it is correct and have the pair step away from game play.

##### Slide Creation (20 min)

After all pairs have been matched, provide a link to all participants to a shared Google Slides document. An example document is linked here. Of note, this link is read only. You can create a copy of this presentation by simply clicking “File” → “Make a Copy.” There is also a sample copy of the file in the supplemental materials.

Have learners find the slide that matches their diagnosis and create a summary slide that reviews image mechanism, epidemiology, imaging findings, and management pearls. Learners can create multiple slides if necessary. Learners can use any resource they have access to but should be encouraged to use appropriate references. It is essential that learners all complete their slides within 20 minutes and that laptops are closed after the slides are complete.

##### Fracture Review (25 min)

After all presentation slides are completed, refresh the shared slide deck to make sure all changes are updated. Have each pair of learners come to the front of the room, present their slide, and take questions from the audience regarding each injury. After all slides are reviewed, email the final slide deck out to participants to use as reference in the future.

#### Small Group Application Exercise Answer Key (sGAE Answer Key)

Below please find each injury listed in our game and a brief explanation of each injury that should be covered in the group presentation.

##### Posterior Shoulder Dislocation

A posterior shoulder dislocation is an uncommon and easily missed diagnosis, which occurs when the humeral head is displaced posteriorly relative to the glenoid.^4^ Common mechanisms of injury include direct trauma to the anterior portion of the shoulder, fall onto a flexed and adducted arm, or sudden and violent muscle contractions, such as following a seizure or electric shock.^5^ Associated injuries may include fractures of the humeral tuberosity and surgical neck, reverse Hill-Sachs lesions, or reverse Bankart lesions. On exam, patients may present with their arm flexed, adducted, and internally rotated, and they cannot externally rotate their arm.^6^ There is often no obvious deformity on exam. Findings on an AP view x-ray are typically subtle, and it is for this reason that this is a commonly missed diagnosis. Radiographic findings that are indicative of a posterior shoulder dislocation include the lightbulb sign (a circular appearance of the humeral head), the rim sign (>6mm overlap between the medial aspect of the humeral head and the anterior glenoid rim), and the trough line sign (two parallel lines of cortical bone in the medial humeral head).^7^ Management of this dislocation typically includes closed reduction followed by immobilizing the arm either with a shoulder immobilizer or a sling, with the arm in a neutral position.^6^

##### Scapholunate Dissociation

A scapholunate dissociation is caused by a tear of the scapholunate interosseous ligament.^8^ The primary mechanism of injury is trauma resulting in severe hyperextension of the wrist, such as through a fall onto an outstretched arm.^9^ Patients will typically present with pain over the radial side of the wrist, particularly over the scaphoid and lunate bones.^9^ On exam, they will have swelling and tenderness to the wrist, as well as pain with dorsiflexion of the wrist. A PA view will reveal a widened gap between the scaphoid and lunate, greater than 3mm.^9^ Injuries associated with a scapholunate dissociation include Colles’ fracture and non-displaced scaphoid fracture.^10^ Management includes placement of a radial gutter splint or a short arm volar posterior mold, with orthopedic referral for operative repair.^10^

##### Clavicular Fracture

Clavicle fractures have their peak incidence in children and young adults, particularly in males.^11^ These fractures commonly occur as a result of a fall onto the shoulder, in addition to traffic accidents and sports injuries.^12^ Fractures primarily occur in the middle third of the clavicle, followed by the distal third, and lastly, the proximal third.^11^ On exam, patients will have tenderness to direct palpation, as well as decreased range of motion of the shoulder. On the AP view of the bilateral shoulders, a fracture of the middle third of the clavicle is likely to be transverse or oblique.^11^ Although rare, other injuries that can be associated with a clavicle fracture include scapular fracture, rib fracture, scapulothoracic dissociation, or axillary/brachial plexus vascular lesion.^13^ For a nondisplaced fracture, conservative treatment includes pain control and reduction of motion at the fracture site through application of a sling. Emergent referral to orthopedics is indicated for open fractures, skin tenting, and neurovascular compromise. Referral to an orthopedist may also be indicated in elderly patients, and for distal clavicle fractures and complete fracture displacement, comminution, and shortening.^13^

##### Monteggia Fracture

A Monteggia fracture is defined as any ulnar fracture that is associated with a radial head dislocation. It may be caused indirectly by hyperpronation of the hand and hyperextension of the elbow, or directly through a blow to the posterior aspect of the ulna, similar to a nightstick fracture.^14^ On exam, a patient with a Monteggia fracture will have limited range of motion of the elbow secondary to pain, as well as an obvious deformity to the affected forearm. Thorough neurovascular exam is important since it is associated with radial nerve injury.^15^ Imaging will demonstrate a fracture of the ulna and dislocation of the radial head, although the radial head dislocation can be easily missed.^16^ In the ED, a Monteggia fracture may be stabilized through reduction of the radial head and splint application with the arm in 90 degrees of flexion. However, urgent referral to orthopedics is ultimately required for open reduction and internal fixation.^15^

##### Galeazzi Fracture

A Galeazzi fracture is a transverse fracture of the distal radius with dislocation of the distal radio-ulnar joint.^17^ It is typically the result of forceful axial loading with the forearm in pronation.^18^ On exam, patients may present with pain, swelling, and deformity to the forearm, and they are likely to have point tenderness over the fracture site. It is important to test forearm pronation and supination for instability.^17^ Associated injuries with a Galeazzi fracture include forearm compartment syndrome and anterior interosseous nerve palsy.^19^ AP and lateral radiographs of the wrist and forearm are recommended and will reveal the fracture to the distal radius. Instability of the distal radio-ulnar joint is suggested by widening of the joint on the AP view, volar or dorsal displacement on the lateral view, an ulnar styloid fracture, or radial shortening that is greater than 5mm.^19^ A long arm splint may be applied, but these fractures ultimately require orthopedic referral for open reduction and internal fixation.^17^

##### Bennett’s Fracture

A Bennett’s fracture is a fracture which occurs at the base of the first metacarpal with associated dislocation of the first carpometacarpal joint.^20^ This fracture occurs when an axial load is placed on a partially flexed metacarpal. This can be the result of a fall onto an extended or abducted thumb or impact onto a clenched fist, such as striking a solid object with a closed fist.^21^ Patients will present with pain and swelling to the thumb base. Exam will be significant for limited range of motion of the first metacarpal and tenderness to palpation of the carpometacarpal joint.^22^ Associated injuries include fracture of an adjacent carpal bone and injury to the ulnar collateral ligament of the thumb metacarpal joint. Imaging will demonstrate a two-piece fracture of the base of the thumb metacarpal.^20^ For a stable Bennett’s fracture, treatment includes closed reduction as needed using thumb traction and metacarpal extension, followed by application of a thumb spica splint and referral to a hand surgeon.^22^ For an unstable fracture and for intra-articular displacement greater than 1mm, the patient must be directly referred to a surgeon.^21^

##### Intertrochanteric Fracture

Intertrochanteric fractures are responsible for approximately one-half of all hip fractures that are caused by a low-energy mechanism, such as a ground level fall.^23^ The risk of incidence of these fractures is increased for females, and with age, history of osteoporosis, and history of prior falls.^24^ On exam, the affected leg will be externally rotated and shortened.^25^ Plain radiographs (AP pelvis, AP and cross table lateral hip, and AP femur) will typically demonstrate a primary fracture line that is oriented from the greater trochanter to the lesser trochanter, with possible comminution and displacement.^23^ Individuals with an intertrochanteric fracture require surgical intervention since a delay in taking them to the OR is associated with higher risk of mortality. Additionally, there is a 20%–30% mortality risk in the first year following an intertrochanteric fracture.^23^

##### Anterior Knee Dislocation

Anterior knee dislocations occur as a result of forced hyperextension, commonly in the case of a motor vehicle accident, as well as in certain sports injuries, such as in football, soccer, and rugby, or by stepping into a hole. On exam, the patient may have a visible and palpable deformity, with limited range of motion of the knee and diffuse pain with palpation of the knee. 26 It is important to assess vascular status of the lower extremity, as popliteal artery injury can occur.^27^ Palsy of the common peroneal nerve occurs in approximately 10%–25% of patients with knee dislocations as well.^28^ X-ray will show a tibia that is anteriorly dislocated from the articular surface of the femur.^28^ An anterior knee dislocation is considered an orthopedic emergency and requires prompt reduction, particularly if there is a pulse deficit. Conservative treatment is advised if the patient is old or sedentary and the joint is stable post-reduction, and includes immobilization for several weeks.^26^,^29^

##### Tibial Plateau Fracture

A tibial plateau fracture occurs when there is a direct forceful blow to the knee, most commonly the lateral knee. Typically, a high energy mechanism of injury, such as a motor vehicle collision or other trauma, is more common in those who are younger, while a low energy mechanism of injury, such as a fall, is more common in the elderly.^30^ On exam, a patient may present with difficulty bearing weight, with possible effusion or stiffness noted to the knee. It is important to assess for emergent findings on exam, such as ligamentous instability, injury to the peroneal nerve, and compartment syndrome.^31^ In a moderate-to-severe fracture, imaging will demonstrate a depression of the lateral tibial plateau on either plain film or CT imaging. Emergent orthopedic consultation is indicated for open fractures, vascular compromise, compartment syndrome, and ligamentous instability.^31^,^32^ For stable cases, a cast or hinged knee brace may be placed, with recommended non-weight bearing and follow up with orthopedics within one week.^33^

##### Maisonneuve Fracture

A Maisonneuve fracture is a combination of a spiral fracture to the proximal fibula, syndesmotic disruption, and injury to the medial ankle, either through disruption of the deltoid ligament or a fracture to the medial malleolus.^10^ It occurs when the ankle is abducted and externally rotated, and is most common in athletes.^34^ A patient will present with pain to the ankle and difficulty bearing weight, with possible ankle edema. It is important to perform an exam of the entire leg since patients may have tenderness over the proximal fibula on exam, although they may only present with pain to the ankle. Peroneal nerve injury may also be associated with a Maisonneuve fracture, highlighting the importance of the neurologic exam as well.^35^ Imaging of the ankle may reveal a fracture to the medial malleolus or widening of the ankle joint, while imaging of the tib fib will reveal a fracture to the proximal fibula.^10^ For stable fractures, a long leg posterior splint may be applied, and an orthopedic consult is advised for possible open reduction and internal fixation of the medial malleolar injury and syndesmotic disruption.^36^

##### Jones Fracture

A Jones fracture is a transverse fracture to the base of the fifth metatarsal.^37^ It occurs when a significant load is applied to the lateral forefoot while the ankle is in plantarflexion. This injury commonly happens in athletes who are involved in sports such as tennis, basketball, or soccer, when the individual is changing direction as their heel is off the ground.^38^,^39^ Patients may present with pain that is diffuse throughout their foot, with difficulty bearing weight. On exam, pain can be localized to the lateral aspect of the foot. Ecchymosis and edema may be noted as well. Imaging will demonstrate a predominantly horizontal fracture that occurs within 1.5cm of the metatarsal tuberosity, at the junction between the diaphysis and metaphysis. If imaging demonstrates a displaced fracture at the proximal diaphysis, or any displacement that is greater than 2mm, the patient must be referred to an orthopedist for possible fixation.^38^,^40^ If the fracture is stable and nondisplaced, conservative treatment is generally appropriate, and consists of immobilization using a short leg posterior splint, with advised non-weight bearing for a minimum of 6 weeks. Jones fractures have a high risk for non-union since the fracture disrupts the already limited blood supply to the junction between the diaphysis and metaphysis. 37,38

##### Lisfranc Injury

The Lisfranc ligament is a major stabilizer of the tarsometatarsal joint, which originates at the first cuneiform and inserts at the medial aspect of the base of the second metatarsal. When an injury occurs to this area, it causes widening of the space between the first and second metatarsal.^41^ This arises either through a crush injury to the foot, or indirect rotational forces and axial load through plantar hyperflexion of the forefoot. Examples include a fall from a height, athletic injury, or motor vehicle accident.^42^ A patient with a Lisfranc injury will present with severe pain to the foot, as well as inability to bear weight. Exam will be significant for ecchymosis, swelling, and pain with palpation along the tarsometatarsal joint.^43^ Associated injuries include proximal metatarsal fracture or tarsal fracture. AP view x-ray will reveal widening of the space between the first and second metatarsals, and between the first and second cuneiform bones.^41^,^42^ If no fracture or dislocation is present, a Lisfranc injury may be treated conservatively with a non-weight bearing cast or boot for 6–8 weeks. If a fracture is present, this is considered an unstable fracture since there is midfoot instability, and emergent orthopedic referral is required for reduction and internal fixation. 42–44

##### Lunate Dislocation

Occurring primarily in young adults, lunate dislocation is the most severe stage of carpal ligamentous injuries. It most commonly results from a traumatic and high energy mechanism of injury, or from a fall onto an outstretched hand.^45^ On exam, a patient will present with significant pain along the dorsum of the wrist, with associated swelling to the wrist and possible deformity. Performing a thorough neurovascular exam is important since median nerve symptoms occur in around 25% of affected patients.^46^ This can be evidenced by loss of two-point discrimination in the distribution of the median nerve. To confirm a lunate dislocation, a 3-view radiograph of the wrist is preferred. The PA view will demonstrate a “piece-of-pie” sign, which is an abnormal triangular appearance of the lunate. The lateral view will demonstrate a “spilled teacup” sign, which is the abnormal volar displacement and tilt of the dislocated lunate that results from the lunate no longer being colinear with the distal radius or the capitate. Because a lunate dislocation is considered unstable, it requires emergent orthopedic consult for reduction and stabilization.^47^

##### Perilunate Dislocation

A perilunate dislocation is a carpal dislocation relative to the lunate, with intact radiolunate articulation. This contrasts with a lunate dislocation, in which the lunate is no longer aligned with the distal radius.^47^ This injury, which constitutes less than 10% of all wrist injuries, most commonly occurs in young adults and is typically the result of increased load onto a hand that is hyperextended and deviated towards the ulna, such as during a fall onto an outstretched hand. On exam, patients will present with pain to the dorsal aspect of the wrist, with swelling, tenderness, and limited range-of-motion noted to the wrist. It is also important to assess neurovascular status on exam since a median nerve injury can occur. 48,49 With regards to imaging, the lateral view will be most indicative of whether a perilunate dislocation is present because it will show dorsal dislocation of the capitate, with the lunate in alignment with the distal radius. The AP view can reveal overlap of the distal and proximal carpal rows, as well as any associated fractures that may be present. 47 Management of this injury includes orthopedic consult for open reduction and internal fixation.^50^
